# Restoring Lost Anti-HER-2 Th1 Immunity in Breast Cancer: A Crucial Role for Th1 Cytokines in Therapy and Prevention

**DOI:** 10.3389/fphar.2016.00356

**Published:** 2016-10-06

**Authors:** Nadia F. Nocera, M. Catherine Lee, Lucy M. De La Cruz, Cinthia Rosemblit, Brian J. Czerniecki

**Affiliations:** ^1^Department of Surgery, University of Pennsylvania Perelman School of MedicinePhiladelphia, PA, USA; ^2^Comprehensive Breast Program, H. Lee Moffitt Cancer CenterTampa, FL, USA

**Keywords:** HER2, immunotherapy, cancer vaccines, breast cancer, Th1 immunity

## Abstract

The ErbB/B2 (HER-2/neu) oncogene family plays a critical role in the development and metastatic spread of several tumor types including breast, ovarian and gastric cancer. In breast cancer, HER-2/neu is expressed in early disease development in a large percentage of DCIS lesions and its expression is associated with an increased risk of invasion and recurrence. Targeting HER-2 with antibodies such as trastuzumab or pertuzumab has improved survival, but patients with more extensive disease may develop resistance to therapy. Interestingly, response to HER-2 targeted therapies correlates with presence of immune response genes in the breast. Th1 cell production of the cytokines interferon gamma (IFNγ) and TNFα can enhance MHC class I expression, PD-L1 expression, augment apoptosis and tumor senescence, and enhances growth inhibition of many anti-breast cancer agents, including anti-estrogens and HER-2 targeted therapies. Recently, we have identified that a loss of anti-HER-2 CD4 Th1 in peripheral blood occurs during breast tumorigenesis and is dramatically diminished, even in Stage I breast cancers. The loss of anti-HER-2 Th1 response is specific and not readily reversed by standard therapies. In fact, this loss of anti-HER-2 Th1 response in peripheral blood correlates with lack of complete response to neoadjuvant therapy and diminished disease-free survival. This defect can be restored with HER-2 vaccinations in both DCIS and IBC. Correcting the anti-HER-2 Th1 response may have significant impact in improving response to HER-2 targeted therapies. Development of immune monitoring systems for anti-HER-2 Th1 to identify patients at risk for recurrence could be critical to improving outcomes, since the anti-HER-2 Th1 response can be restored by vaccination. Correction of the cellular immune response against HER-2 may prevent recurrence in high-risk patients with DCIS and IBC at risk of developing new or recurrent breast cancer.

## Introduction

Cancer immunotherapy has garnered considerable interest, especially in the treatment of HER2/*neu* positive breast cancer. HER2/*neu* (ErbB2) is a protooncogene identified in breast, ovarian, gastric and bladder carcinoma (Ménard et al., 2001). Overexpression of the HER2/*neu* oncodriver typically confers a more aggressive phenotype with a poorer prognosis, especially in breast cancer (BC). Present in approximately 20–25% of invasive breast cancers (IBC) (Meric et al., 2002), overexpression of the HER2 receptor tyrosine kinase is associated with more advanced stage disease at presentation and a rapidly progressive clinical course, including enhanced local-regional extent, early metastatic spread, and resistance to chemotherapy (Pohlmann et al., 2009), all of which contribute to poor clinical outcomes. In cases of *in situ* disease, HER2 overexpression is a powerful predictor of presence of invasion, so *in situ* disease with HER2 positive status is more likely to harbor invasive foci than HER2 negative lesions (Roses et al., 2009).

Understanding the biology of the HER2 oncogene is fundamental to devising and maximizing clinical treatment of the associated BC. HER2 is a type 1 transmembrane protein receptor tyrosine kinase, and when it is overexpressed, it is able to interact with any available receptor tyrosine kinase binding partner, even in the absence of ligand (Elster et al., 2015). This leads to a cascade of downstream signaling in pathways, such as the phosphoinositide-3-kinase pathway, which promote cell growth, proliferation, and metastasis (Subbiah and Gonzalez-Angulo, 2014). Blocking the progression of any these pathways will lead to suppression of HER2 positive disease.

Treatment of HER2/*neu* positive breast cancer has been dramatically improved with specific immunotherapy with monoclonal antibodies including trastuzumab and pertuzumab (O'Sullivan and Smith, 2014; Zanardi et al., 2015). Despite the groundbreaking success of monoclonal antibody treatments, a significant portion of patients develop recurrence after treatment. There is growing evidence that anti-HER2 CD4+ T helper cell (Th1) immunity plays a crucial role in cancer therapy and weak Th1 responses are suggestive of poor treatment response and prognosis (Datta et al., 2016). This role for Th1 immunity is likely due to the fact that CD4+ T helper cells mediate multiple components of both the innate and adaptive immune system response to tumors. Some mechanisms of potential Th1 cell activity include direct cytotoxic tumoricidal activity, modification of antitumor cytokine responses and potentiation of long term immunologic memory (Cintolo et al., 2012). Therefore, recognition of an absent or deficient CD4+ Th1 response may predict patients at risk for treatment failure and poor prognosis, but also correction of an inadequate CD4+ Th1 immune response with the use of anti-HER2 dendritic cell (DC) vaccines could consequently improve response to breast cancer therapy and be an important step in prevention of recurrence.

## Evidence for Anti-HER-2 immune response in tumorigenesis

Humoral and cellular response has been demonstrated in BC. The humoral immune response is sensitized to a specific antigen and drives adaptive immunity, where memory B cells secrete targeted antibodies, and cytotoxic CD8+ T lymphocytes and helper CD4+ T lymphocytes are recruited. In HER2 overexpressed cancers, this dual response permits recognition and destruction of tumor cells. A large retrospective case-control study demonstrated that patients with high levels of auto-antibodies against HER2 have a decreased risk of developing both ductal carcinoma in-situ (DCIS) and IBC (Tabuchi et al., 2016). Healthy women had a significantly higher level of HER2 auto-antibodies than patients with breast cancer of any subtype. However, this study found that some patients with IBC, but not in DCIS, had very high levels of HER2 auto-antibodies, suggesting that B cells in these women are reactivated by breast cancer cells that antigen presenting T cells are able to access easily (Tabuchi et al., 2016).

In addition to the anti-HER2 antibody responses observed, cellular immune responses have been identified as a potential prognostic and predictive indicator in HER2 overexpressed breast cancers. Cellular immunity is mediated by cytotoxic CD8+ T cells and helper CD4+ T cells. CD8+ T cells have the ability to recognize tumor associated antigens (TAA) with major histocompatibility complex (MCH) class I, producing IFN-γ, and inducing cell cycle inhibition, apoptosis and macrophage tumoricidal activity (Mahmoud et al., 2011). CD4+T helper cells complement the activity of CD8+ T cells, by preventing tolerance and promoting the survival of the effector and memory CD8+ cells (Bos and Sherman, 2010). The cytokines IL-2, TNF-α, and IFN-γ, produced by CD4+ Th1 cells, play a crucial role in enhancing CD8+ T cell proliferation and protease expression (Bos and Sherman, 2010). Similarly, tumor antigen specific CD4+ Th1 cells attracted to the tumor microenvironment will secrete IL-2, TNF-α, and IFN-γ, creating an environment for enhanced activity of antigen presenting cells (APC) (Cohen et al., 2000). Patients with advanced breast cancers have been found to have a significantly reduced development of T cells into Th1 cells, resulting in decreased production of Th1 cytokines (Verma et al., 2013). These patients tend to have a poorer prognosis and diminished response to neoadjuvant chemotherapy (Verma et al., 2013).

CD4+ Th1 cell cytokines directly induce senescence, a state of permanent tumor growth arrest and regression *in vivo* (Braumüller et al., 2013), while also being able to induce tumor cell apoptosis *in vitro* (Datta et al., 2015c). Th1 cytokines will shut down angiogenesis and chemokine expression, resulting in sustained tumor regression upon oncogene inactivation (Rakhra et al., 2010; Tkach et al., 2012). Thus, oncogene inactivation appears to induce senescence and apoptosis and activates the immune system, while at the same time; the immune system may also be inducing senescence and apoptosis. Based on this idea, combined treatments of breast cancer cell lines *in vitro* with Th1 cytokines TNF-α and IFN-γ may cause oncogene inactivation of HER2 and subsequent senescence and apoptosis (Braumüller et al., 2013; Namjoshi et al., 2016). Similarly, in a murine model of melanoma, T-cell cytokines IFN-γ and TNF-α synergized with vemurafenib, a BRAF oncogene inhibitor, to induce cell-cycle arrest of tumor cells *in vitro* (Acquavella et al., 2015), underscoring the importance of CD4+ T cell mediated immunity against cancer cells.

## Increased tumor infiltrating lymphocytes improve prognosis in HER2 positive breast cancer

Tumor infiltrating lymphocytes (TIL) have been demonstrated to have an important role in the natural progression and prognosis of many solid cancers, including breast cancer. TILs have been more frequently found in highly proliferative triple negative breast cancer (TNBC), and, to a lesser extent, HER2 positive BC (Perez et al., 2016). Presence of TILs in the tumor stroma suggest a dynamic interaction between the immune system and tumor cells, where cytotoxic T-lymphocytes (CTL) are recognizing and lysing some tumor cells, while others may be escaping immunosurveillance and going on to proliferate by evading the immune system. The term “immunoediting” describes this process. Despite the dynamics of immunoediting within the tumor, it has been recognized that higher levels of TILs correlate to a better prognosis (Mahmoud et al., 2011; Morita et al., 2016; Perez et al., 2016). Specifically, high TIL levels in tumor tissue is associated with increased pathologic complete response (pCR) after chemotherapy as well as improved disease free and overall survival (Wang et al., 2016). One study demonstrated that lymphocyte predominant HER2 positive breast cancer (consisting of at least 60% stromal TILs) has better recurrence free survival when treated with chemotherapy alone, but not with concurrent trastuzumab plus chemotherapy treatment or trastuzumab treatment alone (Perez et al., 2016). In a secondary analysis of the NeoALTTO Trial (Neoadjuvant Lapatinib and/or Trastuzumab Treatment Optimization), the presence of TILs at diagnosis was found to be a positive prognostic indicator independent of which anti-HER2 agent was given with standard chemotherapy (Salgado et al., 2015a). Furthermore, spontaneous healing and regression of disease has been found in HER2 positive DCIS with high TIL (Morita et al., 2016).

The strongest predictor of pCR has been found to be in the level of CD8+ T-lymphocytes (Wang et al., 2016). The presence of CD4+ regulatory T cells (Treg) have not shown a definitive correlation, while higher levels of other CD4+ T cells, such as Th1 cells, are associated with better prognosis (Salgado et al., 2015b). Although cytotoxic CD8+ T cells are a crucial component of cell-mediated immunity, CD4+ T cells maintain a crucial role in recruiting, activating and regulating adaptive immunity. The role of CD4+ T cells in the prognostic value of TIL has been demonstrated in TNBC, where the combination of high CD4+ TIL along with high CD8+ TIL can predict positive clinical outcomes (Matsumoto et al., 2016). Thus, increasing a BC patient's ability to mount an immune response with treatments such as DC vaccination will subsequently increase in CD8+ and CD4+ T cells in the tumor stroma, allowing for increase in pCR.

## Critical role of HER2 in DCIS

DCIS is frequently seen in conjunction with invasive BC and is a presumed precursor to some invasive tumors. Like invasive disease, these lesions are highly heterogeneous and have variable morphology, clinical presentation, and receptor expression (Harada et al., 2011). Interestingly, DCIS lesions with HER2 overexpression are more likely to be associated with invasive disease than HER2 negative lesions (Harada et al., 2011). The overexpression of HER2 in DCIS associated with IDC suggests lesions in evolution and may be a fleeting characteristic (Roses et al., 2009). For example, HER2 may actually be upregulated as an *in situ* tumor evolves to invasive disease, but then is downregulated once a more advanced stage is reached (Roses et al., 2009). HER2-positive disease is a highly proliferating BC subtype (Dieci et al., 2016), supporting a genomic instability theory of HER2-positive DCIS progression to invasive disease.

HER2 expression in DCIS may also lead to HER-2 negative IBC. HER2 positive DCIS is generally seen in association with HER2/neu negative IDC (Harada et al., 2011; Hassett et al., 2016). Here, the immune response against immunogenic HER2 positive cell will occur, thereby allowing cancer cells with HER-2 negative phenotypes to emerge. This “immunoediting” likely accounts for the discrepancy between the rate of HER2 overexpression in DCIS compared to IBC, where is it expressed in up to 56% of DCIS lesions and 20-30% of IBC (Witton et al., 2003; Harada et al., 2011). Although, HER2 positive BC is noted to be an aggressive phenotype, HER2 positive IBC may be balanced by immune surveillance against early stage, HER2 positive disease (Ménard et al., 2001).

## Loss of Anti-HER2 Th1 immunity in breast cancer

CD4+ T helper cells exhibit a profound effect in initiating and maintaining anti-tumor immunity: secretion of Th1 cytokines such as IL-2 and IFN-γ promote CD8+ cytotoxic and natural killer cell function (Kim and Cantor, 2014), as well as induction of MHC class II molecule expression on tumor cells (Mortenson and Fu, 2014). In this fashion, insufficient CD4+ Th1 cell response may facilitate the natural progression of tumor cells.

An existing anti-HER2 Th1 response in healthy donors reflects immune recognition of the HER2 molecule. HER2 is prominently expressed during ductal outgrowth and terminal end bud development in the breast, as well as in branching breast ductal cells during pregnancy and lactation (Eccles, 2011). This pre-sensitization to HER2 in healthy donors likely confers protection against tumorigenic events (Datta et al., 2015c). Suppression of this immune response by the tumor allows evasion of immune surveillance and successful HER2 positive tumorigenesis.

**Figure 1:**
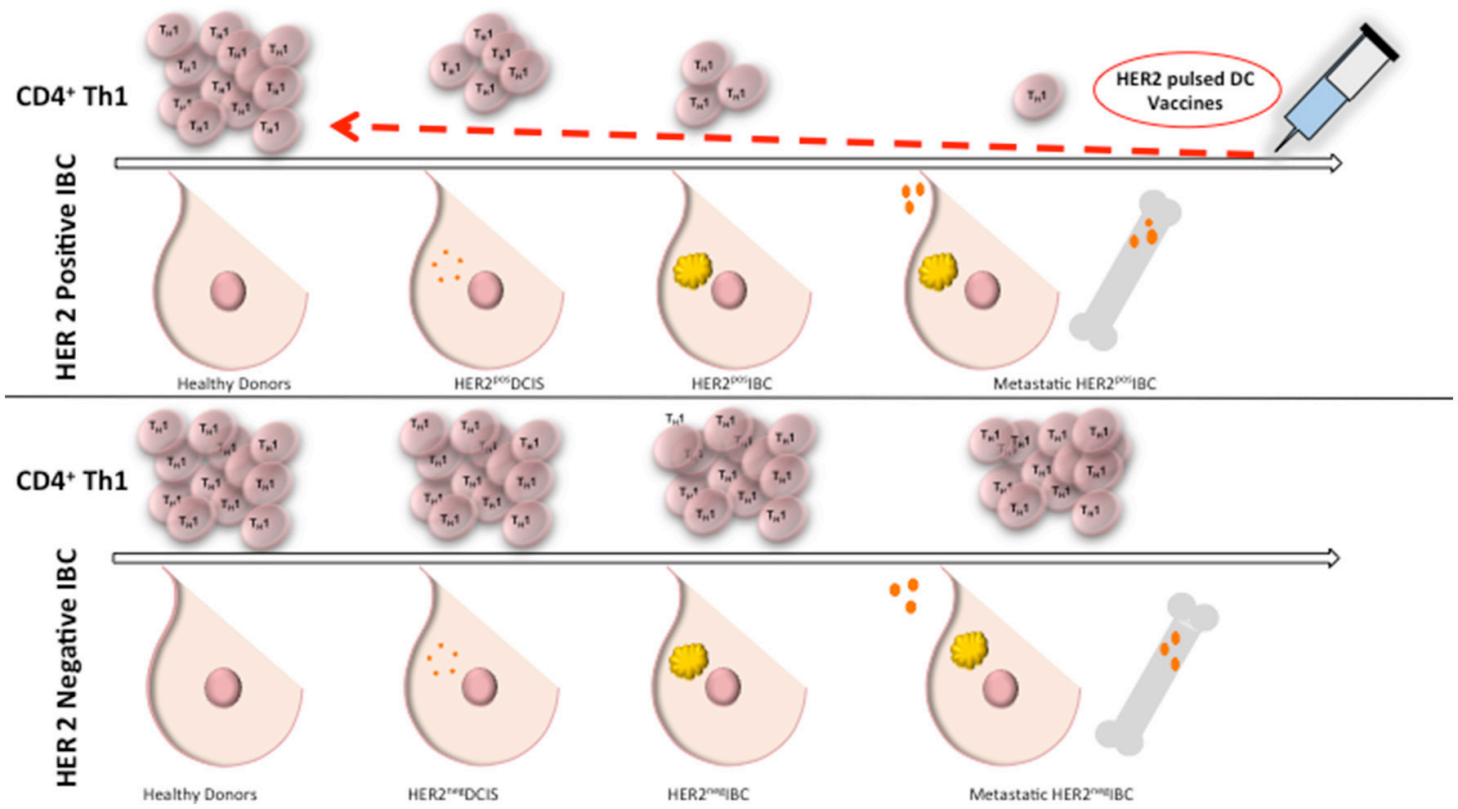
**Anti-HER2 CD4+ Th1 immunity remains intact in healthy donors and in HER2 negative IBC**. Compared to healthy donors, patients with DCIS have a deficient anti-HER2 CD4+ Th1 immunity and there is a nearly absent response in patients with IBC. Patients with HER2 negative BC maintain anti-HER2 Th1 response. Administration of dendritic cell vaccines sensitized to HER2 may restore anti-HER2 Th1 immunity close to the levels of healthy donors. Th1, T helper 1 cells; IBC, invasive breast cancer; DC, dendritic cell; DCIS, ductal carcinoma *in situ*.

There is a progressive loss of anti-HER2 Th1 immunity in HER2 positive BC, where healthy donors and patients with HER2 negative BC have preserved HER2-specific Th1 response, compared to a deficient Th1 response in patients with HER2 positive DCIS, and a nearly absent response in women with HER2 positive IBC (Datta et al., 2015c; Figure 1). Patients with advanced stages of IBC with lymph node positive disease continue to have a further reduction in Th1 response (Zhu et al., 2014). There is evidence of a loss of anti-HER-3 CD4 Th1 responses in triple negative and ER positive breast cancer suggesting loss of anti-oncodriver Th1 responses may be a broader defect than just that occurring in HER-2 tumorigenesis (Fracol manuscript accepted). This loss of anti-oncodriver Th1 mediated immunity may reflect various mechanisms by which tumor cells have succeeded in evasion of the immune response, leading to proliferation and poor response to therapy.

Poor Th1 response may be explained by T cell inhibition, either by T-cell tolerance to tumor antigens (PD-1 and CTLA-4 pathways) or increased pro-apoptosis of CD4+ T cells (Fas pathway). Co-inhibitory signals such as PD-1/PD-L1 and CTLA-1 may provide a contributory role to this loss of immunity. Under normal circumstances, the PD-l/PD-L1 and CTLA-4/B7 checkpoint pathways prevent aberrantly activated T cells from causing autoimmunity; therefore, expression of these ligands by tumor cells allows circumvention of the immune system. PD-1 is expressed on activated T cells, B cells, dendritic cells (DC), natural killer cells and activated monocytes, while and its ligand, PD-L1, is expressed on many cells and tissues such as T cells, natural killer cells and in immune privileged sites (Ge et al., 2013). PD-1 interaction with its ligand, PD-L1, aberrantly expressed on tumor cells, results in T cell inactivation and apoptosis and inhibited activation of tumor antigen-specific T cells (Rosenblatt et al., 2012) by reducing the production of IL-2, IFN-γ, and stimulating IL-10 production (Ge et al., 2013).

Similar to the PD-1 pathway, the CTLA-4 checkpoint pathway also may be utilized by tumor cells expressing the CTLA-4 surface receptor to counteract the activity of T stimulation (Katz et al., 2014). The CTLA-4 receptor competitively inhibits the activity of the T cell co-stimulatory receptor, CD28, therefore impairing tumor-reactive T cells (Pardoll, 2012). Furthermore, CTLA-4 inhibits CD28 co-stimulation by downregulating CD80 and CD86 receptors on dendritic cells (Datta et al., 2015a). Blocking the PD-1/PDL-1 and CTLA4/CD28 pathways with monoclonal antibodies (mAb) may restore antigen presenting cell function, improve T cell function (resulting in increased IFN-γ and TNF-α production), and promote T cell proliferation and T cell targeting of tumors (Ge et al., 2013).

Type I CD4+ T cells are more sensitive to activation-induced cell death via a pro-apoptotic mechanism after chronic antigen stimulation with MHC/peptide complex (Hamad and Schneck, 2001; Wesa et al., 2014). In the progression from DCIS to IBC, T cells progressively encounter increasing stimulation from the presence of the HER2 antigen on tumor cells, so as disease progresses, CD4+ T cells are more likely to undergo activation-induced cell death from continued stimulation and activation with MHC/peptide complexes. This preferential death mechanism has been demonstrated in the CD4+ T cells of melanoma patients with advanced disease. Circulating tumor-associated antigen specific CD4+ T cells in patients with advanced melanoma were found to be more likely to undergo pro-apoptotic programming (Wesa et al., 2014). This pro-apoptotic mechanism is likely due to upregulation of the Fas ligand (FasL) on chronically activated T cells, which normally helps to maintain lymphocyte homeostasis via activation-induced cell death of activated and autoreactive cells (Saff et al., 2004). The constant tumor burden challenge on effector T cells increases FasL expression, and subsequent activation-induced T cell apoptosis (Hoffmann et al., 2002), with an overall effect of weaker anti-tumor and anti-HER2 Th1 immunity.

Tumor-induced overexpression of Fas has been demonstrated in various types of malignancies (Ferrarini et al., 1999; Gastman et al., 1999; Kume et al., 1999; Cheng, 2006), and may be a mechanism of cancer-driven immune evasion, accounting for increased apoptosis seen in tumor infiltrating lymphocytes. Specifically, upregulation of the Fas/FasL death pathway has been exhibited in breast cancer, where upregulation of FasL expression was seen with an increased apoptosis level of tumor infiltrating lymphocytes (Cheng, 2006). Patients with circulating breast cancer cells have been shown to have a significant increase in Fas+ CD4+ T helper cells (Gruber et al., 2013), opening the door for immune escape and worsening disease prognosis. Interestingly, a preclinical murine lymphoma model eliminating Fas or FasL expression on mouse T cells caused the T cells to have enhanced *in vivo* efficacy against tumor cells by increasing T cell survival (Saff et al., 2004). Hence, targeting of the Fas/FasL pathway may be one mechanism to increase anti-HER2 immunity in Th1 cells.

## Treg cells influence antitumor immunity

A tumor-induced immunosuppressive environment may also contribute to loss of immune recognition of HER2 positive BC cells. In addition to overexpression of cell checkpoint pathway peptides and pro-apoptotic peptides, an immunosuppressive network may be created with the use of regulatory T cells (Treg). Normally, Treg cells prevent autoimmunity, but in a tumor microenvironment, Tregs may be recruited, differentiated, expanded and activated to become a tumor Treg and potentially disrupt anti-tumor immunity (Ha, 2009). Naïve T cells may develop into inducible Treg (iTreg) under the influence of various cytokines, such as TGFβ or IL-10 into FoxP3+ Treg cells. FoxP3+ Treg cells may then accumulate in the tumor stroma, providing a barrier to immunosurveillance (Ha, 2009). It has been observed that patients with large, locally advanced breast cancers have increased prevalence of Treg in the peripheral blood and tumor microenvironment (Liyanage et al., 2002; Verma et al., 2013). A higher percentage of the FOXP3+ Tregs in the bloodstream is correlated to poor pathological response to neoadjuvant chemotherapy (Verma et al., 2013). BC patients who are poor clinical responders to neoadjuvant chemotherapy with high FOXP3+ Tregs also have a significant reduction in the production of IFN-γ and TNF-α, consequently creating a pronounced reduction in the Th1 cell profile (Verma et al., 2013).

Recent evidence has suggested that Treg cells are increased in breast cancer models and depletion or inhibition of these Treg cells may improve anti-tumor immunity (Viehl et al., 2006; Hong et al., 2010). Depletion of Treg, either alone or in combination with cancer vaccination, results in an increase in tumor-specific effectors along with slowed tumor growth and improved survival (Viehl et al., 2006). It has been demonstrated that when T cells are stimulated in the absence of Tregs, there is improved tumor rejection via direct lysis and/or production of IFN-γ (Casares et al., 2003). Furthermore, tumors induced in mice develop more slowly and with less frequency when the mice are depleted of Tregs (Gallimore and Godkin, 2008).

The use of DC vaccination has been shown to decrease the prevalence of Treg by depletion or inhibition and conversion to Th1-like effector cells. In one study, CpG-ODN stimulated DC vaccination exhibited pro-inflammatory function and resulted in a decrease in FOXP3+ Tregs in mice (Majumder et al., 2014). It has been demonstrated that FOXP3+ Tregs are inhibited via signaling through Toll-like receptors (TLR), such as TLR-2, TLR-4, TLR-8, and TLR-9 (Lee et al., 2013). This signaling strategy may be harnessed by using TLR4-activated mature DCs to inhibit Treg effects as well as convert the regulatory cells into Th1 cells (Lee et al., 2013). In the presence of immature DCs, Tregs will inhibit effector T cell proliferation, but DCs activated and matured with TLR4 will secrete soluble factors that will release effector cells from suppression by Tregs (Lee et al., 2013). In addition, Tregs deactivated by DCs may contribute to improved immunity by conversion into IFN-γ secreting effector cells (Lee et al., 2013). The effect of DCs on Treg function and conversion into Th1 cells provides another dimension to their importance in the role of antitumor immunity and restoration of Th1 cell function.

## HER2 targeted therapy and resistance

Before the use of monoclonal antibodies in the treatment of cancer, HER2+ breast cancer conferred a poor prognosis. With the advent of trastuzumab, and more recently, pertuzumab, HER2+ breast cancer has a markedly improved overall survival in early and advanced stage disease.

Trastuzumab (Herceptin) is a humanized, recombinant monoclonal antibody (mAb) that binds the extracellular domain of HER2, resulting in its internalization and degradation with subsequent downregulation of downstream PI3K pathway signaling and mediators of cell cycle progression (Yakes et al., 2002). Trastuzumab has antiangiogenic effects and lowers the proapoptotic threshold for chemotherapy (Kumar and Yarmand-Bagheri, 2001), heralding an increase in overall survival when given in combination with chemotherapy to patients with all stages of HER2 positive BC.

Pertuzumab (Perjeta) is another anti-HER2 mAb, similar to trastuzumab, approved in 2014 for in clinical use. Pertuzumab binds to a distinctly different epitope on the extracellular domain of HER2 than trastuzumab, blocking extracellular dimerization of HER2 and HER3 (Gajria and Chandarlapaty, 2011), resulting in complementary mechanisms of action (Scheuer et al., 2009; Gianni et al., 2012; Zanardi et al., 2015). In addition to direct binding to HER2, both mAbs induce antibody dependent cell-mediated cytotoxicity (ADCC) and adaptive immune response to the HER2 protein (Yakes et al., 2002; Musolino et al., 2008).

Similarly, lapatinib (Tykerb), a tyrosine kinase inhibitor (TKI), is a small molecule inhibitor against HER2 and is used to treat HER2 positive BC. The mAbs, trastuzumab, and pertuzumab, target HER2 present in the extracellular domain; lapatinib targets HER2 and EGFR receptor kinases from the intracellular domain to prevent downstream signaling from the HER2/neu and epidermal growth factor receptor pathways (EGFR) (Pohlmann et al., 2009). This distinctly different mechanism of anti-HER2 action has led to the use of lapatinib in the trastuzumab-refractory setting (Geyer et al., 2006). Lapatinib is able to overcome trastuzumab resistance due to truncated HER2 receptors, showing improvement in overall survival and pCR, but with only modest benefit (Geyer et al., 2006; Baselga et al., 2012).

Despite improved outcomes with the use of trastuzumab, the duration of response seems to be limited after single-agent therapy in the metastatic setting (Vogel et al., 2002), and a significant portion of patients who initially respond to the therapy and patients with advanced breast cancer develop resistance to the mAb (Gajria and Chandarlapaty, 2011). Failure of the ADCC may occur due to decreased mAb binding, heterogeneous expression of polymorphic receptors on immune cells, diminished tumor antigen expression on tumor cells, and the concentration or reactivity of immune cells in the tumor micro-environment. Adaptive immune response changes may also contribute to trastuzumab resistance, including dysregulation of the downstream PI3K-AKT-mTOR pathway, accumulation of the truncated kinase active p95-HER2, and alternative receptor kinase signaling (Gajria and Chandarlapaty, 2011). Resistant cells may present with deletions, insertions and missense point mutations that influence the function of receptors, adaptor proteins and second messengers, prohibiting trastuzumab from functioning properly (Pohlmann et al., 2009).

One attempt to combat trastuzumab resistance and improve the potency of trastuzumab therapy is the development of an antibody-drug conjugate to deliver cytotoxic therapy to antigen-expressing tumors (Gajria and Chandarlapaty, 2011). Emantasine (DM1) has been conjugated to trastuzumab (TDM-1). The mechanism of action is similar to trastuzumab alone, in that TDM-1 activates ADCC and inhibits PI3K signaling. However, with TDM-1, trastuzumab allows for selective binding to tumor cells overexpressing HER2; once bound, the DM-1 enters the cells and eradicates them by binding to tubulin (Gajria and Chandarlapaty, 2011; Teicher and Doroshow, 2012). T-DM1 is currently approved for treatment of HER2 positive metastatic breast cancer and is being used in neoadjuvant clinical trials.

## HER2 expression and its relation to vaccination response

The ideal response to vaccination to HER2/neu for breast cancer treatment would be long-lived immunity to the tumor associated antigen. Therefore, dendritic cell vaccines, which induce CD4+ T-helper cells into Th1 helper subsets, are a promising approach to implement lasting immunity. As previously mentioned, Th1 cells have high IFN-γ secretion, which is associated with antitumor immunity and enhances the activity of cytotoxic CD8+ lymphocytes (Cintolo et al., 2012). CD4+ Th1 immune response also produces other cytokines such as TNF-α, a mediator of inflammation, and IL-2, which elicits expansion of lymphocyte populations (Cintolo et al., 2012). In an *in vitro* murine breast cancer cell line, it has been demonstrated that the combination of the Th1 cytokines IFN-γ and TNF-α inhibit cell growth, increase apoptosis, and downregulate HER-2 surface receptor expression (Namjoshi et al., 2016). Restoring or enhancing anti-HER2 Th1 immunity with DC vaccination has been shown to last for up to 60 months post-vaccination (Datta et al., 2015c), underscoring the importance of the CD4+ Th1 helper cell response in eliciting long term antitumor immunity via Th1 cytokine production and CD8+ CTL recruitment.

**Figure 2:**
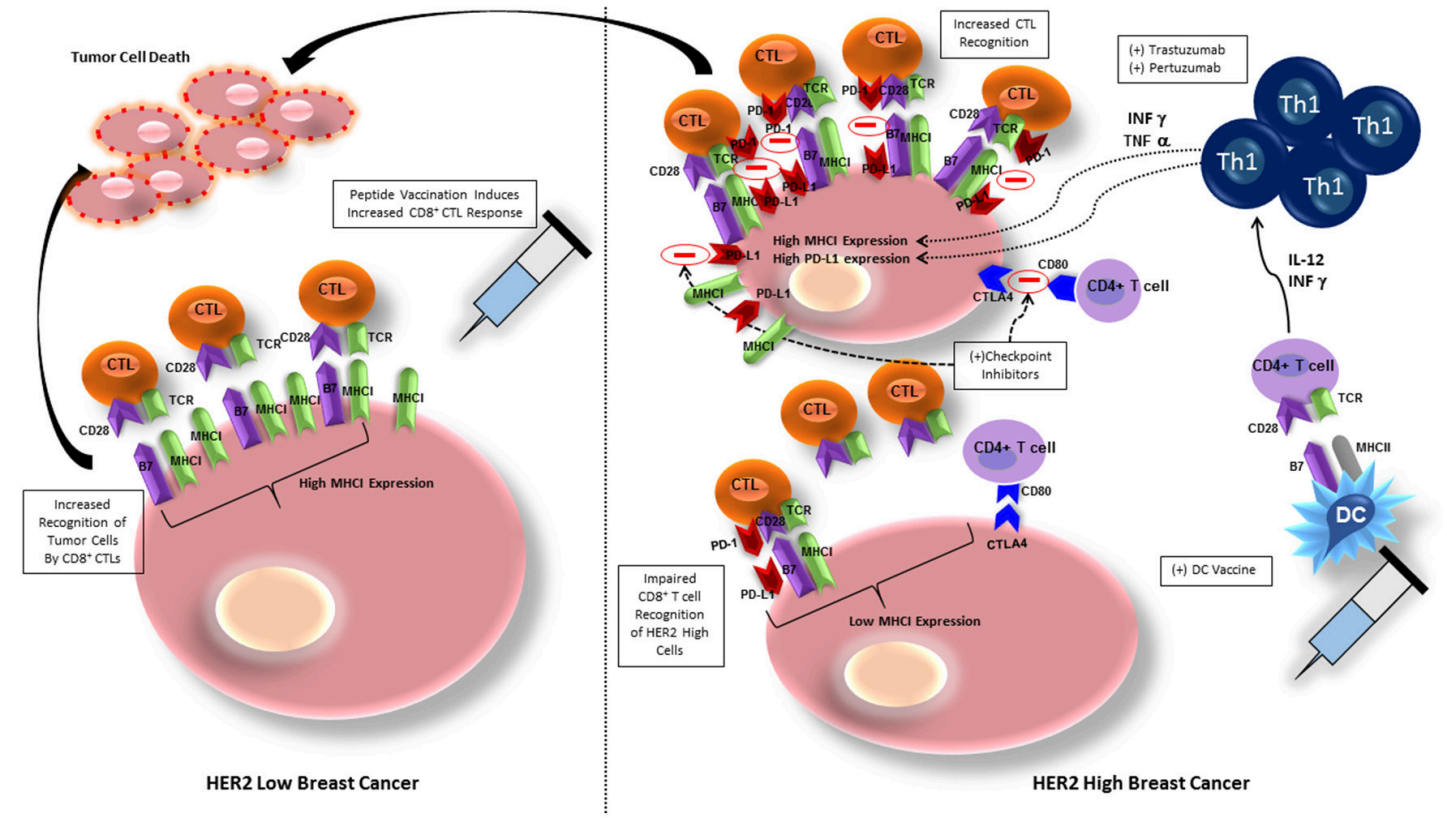
**Breast cancer cells with low HER2 expression exhibit high MHC class I expression and are therefore more easily recognized by CD8+ T cells, allowing for tumor cell killing**. Peptide vaccination elicits CD8+ T cell recognition of BC cells and increases tumor cell lysis. BC cells exhibiting high HER2 expression have downregulation of MHC class I expression, inhibiting CD8+ T cell recognition. BC cells also express PD-L1 and CTLA-4, ligands in cell checkpoint pathways that will inhibit activation of CD8+ and CD4+ T cells, respectively. Administering HER-2 pulsed type I polarized dendritic cell vaccines allows for increased anti-HER-2 CD4+ Th1 cell activation, that secrete IFN-γ and TNF-α, which will upregulate expression of MHC class I, increasing sensitivity to CD8+ CTL mediated lysis. The addition of trastuzumab or pertuzumab to increased IFN-γ and TNF-α expression amplifies MHC class I upregulation. IFN-however increases PD-1L on breast cancer cells that can be blocked by addition of checkpoint inhibitors against PD-L1 and CTLA-4, further maintaining CTL recognition and lysis of tumor cells. CTL, cytotoxic T lymphocyte, MHCI, major histocompatibility complex I; TCR, T cell receptor; DC, dendritic cell; PD-1/PD-L1, programmed cell death protein/programmed cell death protein ligand 1; Th1, T helper cell 1; CTLA-4, cytotoxic T lymphocyte associated protein-4.

The most studied vaccines for HER2 positive breast cancer have been peptide vaccines, more specifically, vaccination against the E75 peptide of HER2/neu. E75 (HER2/neu 369-377) is an immunogenic peptide from the HER2/neu protein that is overexpressed in many breast cancers (Mittendorf et al., 2008). Although, this peptide vaccine has shown some success in stimulating HER2-specific immunity, with lower early recurrence rate than placebo, late recurrences occurred in the vaccinated group due to waning immunity, necessitating vaccine boosters (Mittendorf et al., 2012). Given that the E75 vaccine is a peptide vaccine that elicits a CD8+ cytotoxic T-lymphocyte (CTL) response (Figure 2), immunization with this single HLA class I peptide results in a low-level, short lived response (Knutson et al., 2002). This type of response may be due to lack of activation of other components of the immune system, such as CD4+ T-helper cells.

The E75 peptide vaccine has had the greatest immunologic and clinical benefit in patients with tumors that have low HER2/neu expressing tumors (1+) (Benavides et al., 2009). The benefit seen in vaccination of patients with low HER2/neu expressing tumors may be due to impaired CD8+ cytotoxic T cell recognition of tumor cells that are classically considered HER2 positive (2+/3+). Although, the E75 vaccine increased levels of HER2 specific CD8+ T cells, levels were not strong, and subsequent vaccination boosters were required after levels of CD8+ T cells waned (Mittendorf et al., 2008).

The failure of peptide vaccination to induce HER2-specific CD8+ T cell recognition may be due to overexpression of HER2 causing reduced levels of major histocompatibility complex (MHC) class I, and decreased number of molecules of the antigen processing and presenting machinery (APM), allowing tumor escape from immunosurveillance (Mimura et al., 2011). Associated downregulation of class I expression reduces susceptibility of tumor cells to class I-dependent CD8+ mediated lysis (Datta et al., 2015d; Figure 2). One group attempted to ameliorate the deficiency in CD8+ T cell induction by peptide vaccines by developing a non-replicative vector targeting dendritic cells in a murine model (Tran et al., 2016). By delivering the vaccine with a Shiga Toxin B (STxB) vector, high levels of long-lasting multifunctional, cytotoxic, and high avidity E75-specific CD8+ T cells were obtained. Although the response to the vaccine in combination with a vector was more pronounced, only tumors expressing low levels of HER2 derived the most benefit (Tran et al., 2016).

Targeting of high HER2 expressing tumor cells (2+/3+) is significantly dependent on the cooperation of Th1 cytokines and the trastuzumab-mediated HER2 blockade (Datta et al., 2015d). IFN-γ and TNF-α in combination with trastuzumab and pertuzumab mediate the restoration of MHC class I expression and promotion of HER2-CD8+ T cell targeting against HER2 positive cancers (Datta et al., 2015d; Figure 2). By enhancing induction of Th1 cells, and therefore Th1 cytokines, DC vaccination against HER2 improves this response. *In vitro* studies provide a possible explanation for this synergistic effect—trastuzumab promotes receptor internalization, HER-2 protein degradation, and MHC class I peptide presentation of the vaccinated peptide, thereby increasing tumor lysis (Mittendorf et al., 2006). Indications for trastuzumab are HER2/neu overexpressing tumors with IHC 3+ or FISH ≥ 2.2, node positive, and metastatic breast cancer patients (Vogel et al., 2002). Therefore, when trastuzumab is given for its specified indication, the effect is twofold: (1) humoral immunity against HER2, and (2) induction of Th1 cytokines to promote HER2 positive cancer cell recognition and destruction.

## Immune response in HER2 positive breast cancer can predict outcomes

Until recently, there has been a lack of measurable and modifiable immune signatures available to correlate with the success or failure of neoadjuvant chemotherapy in achieving pCR. Achievement of pCR is one of the strongest predictors of disease control and survival, and patients achieving pCR after neoadjuvant chemotherapy plus anti-HER2 therapy have a more favorable prognosis, with a decrease in recurrence and increase in long-term survival (Untch et al., 2011; Kim et al., 2013). Identifying genomic markers related to treatment response in HER2/neu positive breast cancer is important in detecting patients at risk for tumor recurrence after treatment. Identifiable factors are immune response genes that may be expressed on tumor cells or may be factors expressed and detected in a patient's peripheral blood. The monoclonal antibody trastuzumab is standard treatment for HER2 positive breast cancer, but because some patients have a poor response or relapse after treatment, and recognizing relevant genes corresponding to treatment response is of particular importance. HER2 overexpression in BC is not predictive of either significant response to treatment or risk of relapse. However, identification of other genes correlating to treatment response may direct therapy.

A cohort of immune function genes expressed on tumor cells may predict which patients with HER2 positive breast tumors benefit from adjuvant trastuzumab treatment. Relevant pathways and gene expression signatures on immune-related genes have been found to be associated with treatment response and likelihood of recurrence (Perez et al., 2015; Dieci et al., 2016). The pathways associated with increased recurrence free survival after adjuvant trastuzumab include: cytokine-cytokine receptor interaction, T-cell receptor signaling in CD8+ T cells, interferon gamma pathway, tumor necrosis factor receptor signaling pathway, cell surface interaction at the vascular endothelium, and class I PI3K signaling event pathways (Perez et al., 2015). Whole transcriptome analysis of a HER2 positive breast cancer cohort by Perez et al. demonstrated a strong association between immune gene expression and recurrence free survival following treatment with adjuvant trastuzumab (Perez et al., 2015). Tumors that are positive for these immune function genes show a positive correlation with recurrence-free survival after adjuvant trastuzumab therapy. The trastuzumab risk (TRAR) prediction model is based on expression levels of 41 genes predictive of early relapse (Triulzi et al., 2015). In particular, tumors with low risk of relapse (TRAR-low) had enhanced expression and enrichment of immune associated pathways and, subsequently, a higher immune cell infiltrate in the tumor milieu. These studies support the association between immune gene expression and recurrence free survival, suggesting that a subset of patients with highly immunologic HER2 positive tumors are more likely to benefit from treatment with adjuvant trastuzumab. In the future, patients with low expression of these genes may be identified and given other chemotherapy combinations, in addition to HER2- sensitized dendritic cell vaccines to restore immune response against HER2 positive cancers.

Recently, our group has shown anti-HER2 Th1 response to be a systemic immune correlate to pCR, and may be a sufficient measure of immune monitoring and risk of recurrence in patients with treated HER2 positive IBC (Datta et al., 2015b, 2016). In patients receiving trastuzumab-based neoadjuvant chemotherapy, anti-HER2 CD4+ T cell immunity was dramatically higher in women obtaining pCR, compared to those with residual disease (Datta et al., 2015b). This correlation provides an avenue for estimating pCR after neoadjuvant HER2-directed treatment plus chemotherapy. Patients with a lack of pCR may be due to a depressed anti-HER2 Th1 cell response (Datta et al., 2015b). For example, a pilot study evaluating the anti-HER2 Th1 response in 95 HER2 positive invasive breast cancer patients treated with standard curative therapy suggested that a depressed anti-HER2 Th1 response correlates to increased risk of recurrence (Datta et al., 2015b). Furthermore, it appears as though absent tumor-level Th1 gene expression and deficient circulating anti-HER2 Th1 immunity may predict failure of HER2-targeted therapy (Datta et al., 2016). These results suggest that CD4+ Th1 cells provoke an important antitumor immune response, even when patients are given HER2 targeting agents. Therefore, patients with residual disease after chemotherapy or resistant lesions after HER2 targeted therapy may benefit from restoration of this anti-HER2 response. In patients with depressed anti-HER2 Th1 response and incomplete response to trastuzumab plus chemotherapy, supplementation of treatment with a HER2 targeted dendritic cell (DC) vaccination have been found to drive an increased anti-HER2 response (Datta et al., 2015b; Figure 1). Tracking anti-HER2 Th1 immunity using peripheral blood draws may identify patients at increased risk for recurrence based on fluctuations in Th1 immunity, allowing for much earlier therapeutic intervention (Datta et al., 2016).

## Restoration of Anti-HER2 Th1 immune response

The Th1 response has not only proven to be an immune correlate to pCR, but may also be modified to improve patient outcomes. Anti-HER2 Th1 response has the potential to be restored with the use of HER2-sensitized dendritic cell (DC) vaccines. DCs are pulsed with synthetic HER2 peptides, which, although confer a more selective portion of the protein, have been shown to be better processed by DCs than a whole protein. This improves cross-presentation and activation of CD8+ T cells (Rosalia et al., 2013).

DCs deliver co-stimulatory signals to T cells, stimulating activation and polarization of CD8+ T cells into CTLs and CD4+ T cells into Th1, Th2, and Th17 cells (Kaiko et al., 2008). HER2-pulsed DC vaccination will also boost effectiveness of anti-HER2 immunity by increasing IL-12 production, which increases T cell functionality by polarizing T cells into the INF-γ and TNF-α secreting Th1 phenotype *in vivo*, promoting anti-tumor immunity (Koski et al., 2012). It has been demonstrated in an *in vitro* model of HER2-overexpressing cells that an increase in INF-γ and TNF-α will upregulate expression of MHC class I, therefore increasing sensitivity to CD8+ T cell mediated lysis (Datta et al., 2015d). The dual combination of INF-γ and TNF-α secreted by Th1 cells has been shown to be responsible for the clinical effects of DC vaccination by increasing tumor cell apoptosis, senescence and HER2 receptor downregulation (Namjoshi et al., 2016; Figure 2). The paired cytokines activate caspase-3, which is linked to downstream apoptotic processes starting after only 5 h of cytokine exposure (Namjoshi et al., 2016). Addition of trastuzumab to the combination of INF-γ and TNF-α only amplifies this result, likely due to its inhibitory effect on both the MAPK and PI3K/AKT pathways, allowing INF-γ/TNF-α to potentiate class I upregulation (Datta et al., 2015d; Figure 2).

After administration of DC vaccines to HER2 positive DCIS and stage I breast cancer patients, substantial anti-HER2 Th1 immunity is induced (Figure 1), with pCR rates approaching 25%, with loss of the target antigen in the remainder of patients (Sharma et al., 2012; Datta et al., 2015b). When HER2-pulsed DC vaccines were given to patients with an anti-HER2 Th1 immune deficit in the neoadjuvant setting, anti-HER2 Th1 immunity was restored and observed for up to 60 months post-vaccination (Datta et al., 2015c). This restoration of Th1 immunity is not seen with neoadjuvant trastuzumab and/or chemotherapy (Datta et al., 2015c). Deficient anti-HER2 Th1 immunity is associated with incomplete response to neoadjuvant therapy. Fortunately, even in patients who have had several rounds of pretreatment, loss of Th1 immunity can be rectified with HER2 pulsed DC vaccination (Datta et al., 2015b). DC vaccination, therefore, has the ability to restore and improve anti-HER2 Th1 immunity in the neoadjuvant setting, suggesting a role for combination therapy with DC vaccination to enhance achievement of pCR.

## Opportunities for combining dendritic cell vaccines with standard Anti-HER2 therapy

Although, there have been revolutionary advances in drug development for HER2-directed therapy in breast cancer, a significant portion of treated patients do not achieve pCR, and/or develop resistance to treatment. For example, patients who receive trastuzumab and cytotoxic chemotherapy in the neoadjuvant setting have a 40–60% pCR rate (Gianni et al., 2010; Untch et al., 2011; Kim et al., 2013). Heterogeneity of HER2 overexpression is a primary cause of an incomplete response, but immune responses related to trastuzumab resistance may also contribute to these residual tumors (Datta et al., 2015c). Vaccination against HER2 has been shown to significantly boost and maintain immunity to HER2 in trastuzumab treated patients, allowing the immune system to overcome resistance to HER2 targeted therapy by restoring MHC class I expression on HER2 high tumor cells (Datta et al., 2015d). Increased MHC class I expression therefore induces enhanced killing by HER2-stimulated cytotoxic T lymphocytes (CTL) (Mittendorf et al., 2006; Disis et al., 2009), improving rates of pCR.

Trastuzumab has been evaluated in combination with anti-HER2 vaccination more than other newer agents with similar functions, such as pertuzumab and TKIs such as lapatinib. Although pertuzumab has not yet been studied in combination with vaccinations, its similar mechanism of action to trastuzumab likely will lend itself to similar results when administered concurrently with DC vaccines. A phase I trial of lapatinib and HER2 vaccination has been tested and another TKI, ataxinib, has already demonstrated improved therapeutic efficacy when combined with anti-HER2 DC vaccination in a preclinical model of murine melanoma, opening the door for further studies (Bose et al., 2012; Hamilton et al., 2012).

Blockade of checkpoint pathways may also allow improvement in efficacy of targeted immunotherapies, such as DC vaccination. PD-1/PD-L1 signaling can inhibit DC maturation, as well as decrease the interaction between DCs and T cells, so abrogation of this pathway would improve subsequent immune response (Ge et al., 2013). In a murine model of human breast cancer, blockade of the PD-1/PD-L1 pathway using a monoclonal antibody yielded improved therapeutic efficacy of DC vaccination with prolonged survival and prevention of tumor growth (Ge et al., 2013). Similarly, when DC vaccination was combined with aOX40 (anti-CD134)/aCTLA-4 (anti–cytotoxic T-lymphocyte–associated protein 4) monoclonal antibody in a mouse mammary carcinoma model, overall survival was significantly improved (Linch et al., 2016). The combination of aOX40 and DC vaccination increased CD8+ T cell specificity and response, as well as promoted a robust Th1 cell response as evidenced by increased levels of IFNγ, TNFα, and IL-2 production (Linch et al., 2016). *In vitro* studies have also shown that synergy between the Th1 cytokines IFN-γ and TNF-α, which have increased production after DC vaccination, and trastuzumab induces PD-L1 expression and MHC class I upregulation on HER2- overexpressing cells *in vitro*, facilitating CD8+ T cell recognition of tumor cells (Datta et al., 2015d; Figure 2). Therefore, given in combination with checkpoint inhibitors, DC vaccination provides a synergistic promotion of Th1 response and increases the frequency of tumor infiltrating CD4+ and CD8+ T cells (Linch et al., 2016), ultimately providing an improved prognosis.

## Future outlook

In the evolving field of cancer immunotherapy, the HER2/neu tumor associated antigen has developed a central role. Monoclonal antibodies against this antigen have yielded considerable success in treating HER2 positive breast cancer, but resistance to this therapy is a significant limitation. Identification of a loss of anti-HER2 CD4+ Th1 response in tumorigenesis, and its correlation to a poor response to traditional traztuzumab plus chemotherapy, have paved a path toward the potential for restoration of this response with anti-HER2 dendritic cell vaccination therapy. The use of DC vaccination in combination with traditional HER2-directed therapy has yielded promising results due to reestablishment of Th1 immunity, resulting in increased production of Th1 cytokines and subsequently improved patient outcomes. Th1 cytokines maintain a crucial role in the tumor microenvironment by enhancing MHC class I expression, PD-L1 expression, apoptosis, and tumor senescence therefore restoring IFN-γ through systemic administration offers opportunity to enhance effectiveness of standard chemotherapy and HER-2 directed therapy. Monitoring levels of anti-HER2 Th1 responsivity may help identify vulnerable patient populations at risk for treatment failure provide means for early intervention by vaccination for immune restoration. Monitoring and correcting the cellular immune response against HER2 may prevent recurrence in high-risk patients with DCIS and in IBC. Elucidating this critical role for anti-HER2 Th1 immunity has shaped the landscape of breast cancer immunotherapy, and will further prove to be an essential component of future breast cancer treatment.

## Author contributions

NN: conception and design, acquisition of data, writing/drafting manuscript, revising for important content, final approval of version to be published agreement for accountability of published material; ML: writing/drafting manuscript, revising for important content, final approval of version to be published; agreement for accountability of published material; LD: revising for important content, final approval of version to be published; agreement for accountability of published material; CR: writing/drafting manuscript, final approval of version to be published; agreement for accountability of published material; BC: conception and design, acquisition of data, writing/drafting manuscript, revising for important content, final approval of version to be published; agreement for accountability of published material.

## Disclosures

No external funding was secured for this study. The authors have no financial relationships or conflicts of interest relevant to this article to disclose.

### Conflict of interest statement

The authors declare that the research was conducted in the absence of any commercial or financial relationships that could be construed as a potential conflict of interest.

## References

[B1] Acquavella N. Clever D. Yu Z. Roelke-Parker M. Palmer D. C. Xi L.. (2015). Type I cytokines synergize with oncogene inhibition to induce tumor growth arrest. Cancer Immunol. Res. 3, 37–47. 10.1158/2326-6066.CIR-14-0122 25358764 PMC4289107

[B2] Baselga J. Bradbury I. Eidtmann H. Di Cosimo S. de Azambuja E. Aura C.. (2012). NeoALTTO Study Tream. Lapatinib with trastuzumab for HER2-positive early breast cancer (NeoALTTO): a randomised, open-label, multricentre, phase 3 trial. Lancet 379, 633–640. 10.1016/S0140-6736(11)61847-3 22257673 PMC5705192

[B3] Benavides L. C. Gates J. D. Carmichael M. G. Patil R. Holmes J. P. Hueman M. T.. (2009). The impact of HER2/neu expression level on response to the E75 vaccine: from U.S. Military Cancer Institute Clinical Trials Group Study I-01 and I-02. Clin. Cancer Res. 15, 2895–2904. 10.1158/1078-0432.CCR-08-1126 19351776

[B4] Bos R. Sherman L. A. (2010). CD4+ T-cell help in the tumor milieu is required for recruitment and cytolytic function of CD8+ T lymphocytes. Cancer Res. 70, 8368–8377. 10.1158/0008-5472.CAN-10-1322 20940398 PMC2970736

[B5] Bose A. Lowe D. B. Rao A. Storkus W. J. (2012). Combined vaccine+axitinib therapy yields superior antitumor efficacy in a murine melanoma model. Melanoma Res. 22, 236–243. 10.1097/CMR.0b013e3283538293 22504156 PMC3340498

[B6] Braumüller H. Wieder T. Brenner E. Aßmann S. Hahn M. Alkhaled M.. (2013). T-helper-1-cell cytokines drive cancer into senescence. Nature 494, 361–365. 10.1038/nature11824 23376950

[B7] Casares N. Arribillaga L. Sarobe P. Dotor J. Lopez-Diaz de Cerio A. Melero I.. (2003). CD4+/CD25+ regulatory cells inhibit activation of tumor-primed CD4+ T cells with IFN-gamma-dependent antiangiogenic activity, as well as long-lasting tumor immunity elicited by peptide vaccination. J. Immunol. 171, 5931–5939. 10.4049/jimmunol.171.11.5931 14634104

[B8] Cheng B. (2006). Association between up-regulation of Fas ligand expression and apoptosis of tumor-infiltrating lymphocytes in Human Breast Cancer. J. Huazhong Univ. Sci. Technol. Med. Sci. 25, 573–575. 10.1007/s11596-006-0524-5 17219972

[B9] Cintolo J. A. Datta J. Mathew S. J. Czerniecki B. J. (2012). Dendritic cell-based vaccines: barriers and opportunities. Future Oncol. 8, 1273–1299. 10.2217/fon.12.125 23130928 PMC4260651

[B10] Cohen P. A. Peng L. Plautz G. E. Kim J. A. Weng D. E. Shu S. (2000). CD4+ T cells in adoptive immunotherapy and the indirect mechanism of tumor rejection. Crit. Rev. Immunol. 2, 85–95. 10.1615/critrevimmunol.v20.i1.20 10770269

[B11] Datta J. Berk E. Cintolo J. A. Xu S. Roses R. E. Czerniecki B. J. (2015a). Rationale for a multimodality strategy to enhance the efficacy of dendritic cell-based cancer immunotherapy. Front. Immunol. 6:271. 10.3389/fimmu.2015.00271 26082780 PMC4451636

[B12] Datta J. Berk E. Xu S. Fitzpatrick E. Rosemblit C. Lowenfeld L.. (2015b). Anti-HER2 CD4(+) T-helper type 1 response is a novel immune correlate to pathologic response following neoadjuvant therapy in HER2-positive breast cancer. Breast Cancer Res. 17, 71. 10.1186/s13058-015-0584-1 25997452 PMC4488128

[B13] Datta J. Fracol M. McMillan M. T. Berk E. Xu S. Goodman N.. (2016). Association of depressed anti-HER2 T-helper type 1 response with recurrence in patients with completely treated HER2-positive breast cancer: role for immune monitoring. JAMA Oncol. 2, 242–246. 10.1001/jamaoncol.2015.5482 26719971

[B14] Datta J. Rosemblit C. Berk E. Showalter L. Namjoshi P. Mick R.. (2015c). Progressive loss of anti-HER2 CD4^+^ T-helper type 1 response in breast tumorigenesis and the potential for immune restoration. Oncoimmunology. 4:e1022301. 10.1080/2162402X.2015.1022301 26451293 PMC4589053

[B15] Datta J. Xu S. Rosemblit C. Smith J. B. Cintolo J. A. Powell D. J. Jr.. (2015d). CD4(+) T-helper type 1 cytokines and trastuzumab facilitate CD8(+) T-cell targeting of HER2/neu-expressing cancers. Cancer Immunol. Res, 3, 455–463. 10.1158/2326-6066.CIR-14-0208 25791067 PMC4556111

[B16] Dieci M. V. Griguolo G. Miglietta F. Guarneri V. (2016). The immune system and hormone-receptor positive breast cancer: Is it really a dead end? Cancer Treat. Rev., 46, 9–19. 10.1016/j.ctrv.2016.03.011 27055087

[B17] Disis M. L. Wallace D. R. Gooley T. A. Dang Y. Slota M. Lu H.. (2009). Concurrent trastuzumab and HER2/neu-specific vaccination in patients with metastatic breast cancer. J. Clin. Oncol. 27, 4685–4692. 10.1200/JCO.2008.20.6789 19720923 PMC2754913

[B18] Eccles S. A. (2011). The epidermal growth factor receptor/Erb-B/HER family in normal and malignant breast biology. Int. J. Dev. Biol. 55, 685–696. 10.1387/ijdb.113396se 22161825

[B19] Elster N. Collins D. M. Toomey S. Crown J. Eustace A. J. Hennessy B. T. (2015). HER2-family signalling mechanisms, clinical implications and targeting in breast cancer. Breast Cancer Res. Treat. 149, 5–15. 10.1007/s10549-014-3250-x 25542271

[B20] Ferrarini M. Imro M. A. Sciorati C. Heltai S. Protti M. P. Pellicciari C.. (1999). Blockade of the Fas-triggered intracellular signaling pathway in human melanomas is circumvented by cytotoxic lymphocytes. Int. J. Cancer. 17, 573–579. 10225447 10.1002/(sici)1097-0215(19990517)81:4<573::aid-ijc12>3.0.co;2-w

[B21] Gajria D. Chandarlapaty S. (2011). HER2-amplified breast cancer: mechanisms of trastuzumab resistance and novel targeted therapies. Expert Rev. Anticancer Ther. 11, 263–275. 10.1586/era.10.226 21342044 PMC3092522

[B22] Gallimore A. Godkin A. (2008). Regulatory T cells and tumour immunity – observations in mice and men. Immunology 123, 157–163. 10.1111/j.1365-2567.2007.02748.x 18067556 PMC2433304

[B23] Gastman B. R. Atarshi Y. Reichert T. E. Saito T. Balkir L. Rabinowich H.. (1999). Fas ligand is expressed on human squamous cell carcinomas of the head and neck and it promotes apoptosis of T lymphocytes. Cancer Res. 59, 5356–5364. 10537320

[B24] Ge Y. Xi H. Ju S. Zhang X. (2013). Blockade of PD-1/PD-L1 immune checkpoint during DC vaccination induces potent protective immunity against breast cancer in hu-SCID mice. Cancer Lett. 336, 253–259. 10.1016/j.canlet.2013.03.010 23523609

[B25] Geyer C. E. Forster J. Lindquist D. Chan S. Romieu C. G. Pienkowski T.. (2006). Lapatinib plus capecitabine for HER2-positive advanced breast cancer. N. Engl. J. Med. 355, 2733–2743. 10.1056/NEJMoa064320 17192538

[B26] Gianni L. Eiermann W. Semiglazov V. Manikhas A. Lluch A. Tjulandin S.. (2010). Neoadjuvant chemotherapy with trastuzumab followed by adjuvant trastuzumab versus neoadjuvant chemotherapy alone, in patients with HER2-positive locally advanced breast cancer (the NOAH trial): a randomised controlled superiority trial with parallell HER2-negative cohort. Lancet 375, 377–384. 10.1016/S0140-6736(09)61964-4 20113825

[B27] Gianni L. Pienkowski T. Im Y. H. Roman L. Tseng L. M. Liu M. C.. (2012). Efficacy and safety of neoadjuvant pertuzumab and trastuzumab in women with locally advanced, inflammatory, or early HER2-positive breast cancer (NeoSphere): a randomised multicentre, open-label, phase 2 trial. Lancet Oncol. 13, 25–32. 10.1016/S1470-2045(11)70336-9 22153890

[B28] Gruber I. Landenberger N. Staebler A. Hahn M. Wallwiener D. Fehm T. (2013). Relationship between circulating tumor cells and peripheral T-cells in patients with primary breast cancer. Anticancer Res. 33, 2233–2238. 23645781

[B29] Ha T. Y. (2009). The role of regulatory T cells in cancer. Immune Netw. 9, 209–235. 10.4110/in.2009.9.6.209 20157609 PMC2816955

[B30] Hamad A. R. Schneck J. P. (2001). Antigen-induced T cell death is regulated by CD4 expression. Int. Rev. Immunol. 20, 535–546. 10.3109/08830180109045577 11890611

[B31] Hamilton E. Blackwell K. Hobeika A. C. Clay T. M. Broadwater G. Ren X. R.. (2012). Phase 1 clinical trial of HER2-specific immunotherapy with concomitant HER2 kinase inhibition [corrected]. J. Transl. Med. 10:28. 10.1186/1479-5876-10-28 22325452 PMC3306270

[B32] Harada S. Mick R. Roses R. E. Graves H. Niu H. Sharma A.. (2011). The significance of HER-2/neu receptor positivity and immunophenotype in ductal carcinoma in situ with early invasive disease. J. Surg. Oncol. 104, 458–465. 10.1002/jso.21973 21557226 PMC3168675

[B33] Hassett M. J. Jiang W. Habel L. A. Nekhlyudov L. Achacoso N. Acton L.. (2016). Characteristics of second breast events among women treated with breast-conserving surgery for DCIS in the community. Breast Cancer Res. Treat. 155, 541–549. 10.1007/s10549-016-3692-4 26843057 PMC4767622

[B34] Hoffmann T. K. Dworacki G. Tsukihiro T. Meidenbauer N. Gooding W. Johnson J. T.. (2002). Spontaneous apoptosis of circulating T lymphocytes in patients with head and neck cancer and its clinical importance. Clin. Cancer Res. 8, 2553–2562. 12171883

[B35] Hong H. Gu Y. Zhang H. Simon A. K. Chen X. Wu C.. (2010). Depletion of CD4+CD25+ regulatory T cells enhances natural killer T cell-mediated anti-tumour immunity in a murine mammary breast cancer model. Clin. Exp. Immunol. 159, 93–99. 10.1111/j.1365-2249.2009.04018.x 19817769 PMC2802699

[B36] Kaiko G. E. Horvat J. C. Beagley K. W. Hansbro P. M. (2008). Immunological decision-making: how does the immune system decide to mount a helper T-cell response? Immunology 123, 326–338. 10.1111/j.1365-2567.2007.02719.x 17983439 PMC2433332

[B37] Katz T. Avivi I. Benyamini N. Rosenblatt J. Avigan D. (2014). Dendritic cell cancer vaccines: from the bench to the bedside. Rambam Maimonides Med. J. 5, e0024. 10.5041/RMMJ.10158 25386340 PMC4222413

[B38] Kim H. J. Cantor H. (2014). CD4 T-cell subsets and tumor immunity: the helpful and the not-so-helpful. Cancer Immunol. Res. 2, 91–98. 10.1158/2326-6066.CIR-13-0216 24778273

[B39] Kim M. M. Allen P. Gonzalez-Angulo A. M. Woodward W. A. Meric-Bernstam F. Buzdar A. U.. (2013). Pathologic complete response to neoadjuvant chemotherapy with trastuzumab predicts for improved survival in women with HER2-overexpressing breast cancer. Ann. Oncol. 24, 1999–2004. 10.1093/annonc/mdt131 23562929 PMC3718505

[B40] Knutson K. L. Schiffman K. Cheever M. A. Disis M. L. (2002). Immunization of cancer patients with a HER-2/neu, HLA-A2 peptide, p369-377, results in short-lived peptide-specific immunity. Clin. Cancer Res. 8, 1014–1018. 12006513

[B41] Koski G. K. Koldovsky U. Xu S. Mick R. Sharma A. Fitzpatrick E.. (2012). A novel dendritic cell-based immunization approach for the induction of durable Th1-polarized anti-HER-2/neu responses in women with early breast cancer. J. Immunother. 35, 54–65. 10.1097/CJI.0b013e318235f512 22130160 PMC3241864

[B42] Kumar R. Yarmand-Bagheri R. (2001). The role of HER2 in angiogenesis. Semin. Oncol. 28, 27–32. 10.1016/S0093-7754(01)90279-9 11706393

[B43] Kume T. Oshima K. Yamashita Y. Shirakusa T. Kikuchi M. (1999). Relationship between Fas-ligand expression on carcinoma cell and cytotoxic T-lymphocyte response in lymphoepithelioma-like cancer of the stomach. Int. J. Cancer 20, 339–343. 10404082 10.1002/(sici)1097-0215(19990820)84:4<339::aid-ijc1>3.0.co;2-2

[B44] Lee M. K. IV Xu S. Fitzpatrick E. H. Sharma A. Graves H. L. Czerniecki B. J. (2013). Inhibition of CD4+CD25+ regulatory T cell function and conversion into Th1-like effectors by a Toll-like receptor-activated dendritic cell vaccine. PLoS ONE 8:e74698. 10.1371/journal.pone.0074698 24244265 PMC3823870

[B45] Linch S. N. Kasiewicz M. J. McNamara M. J. Hilgart-Martiszus I. F. Farhad M. Redmond W. L. (2016). Combination OX40 agonism/CTLA-4 blockade with HER2 vaccination reverses T-cell anergy and promotes survival in tumor-bearing mice. Proc. Natl. Acad. Sci. U.S.A. 113, E319–E327. 10.1073/pnas.1510518113 26729864 PMC4725491

[B46] Liyanage U. K. Moore T. T. Joo H. G. Tanaka Y. Herrmann V. Doherty G.. (2002). Prevalence of regulatory T cells is increased in peripheral blood and tumor microenvironment of patients with pancreas or breast adenocarcinoma. J. Immunol. 169, 2756–2761. 10.4049/jimmunol.169.5.2756 12193750

[B47] Mahmoud S. M. Paish E. C. Powe D. G. Macmillan R. D. Grainge M. J. Lee A. H.. (2011). Tumor-infiltrating CD8+ lymphocytes predict clinical outcome in breast cancer. J. Clin. Oncol. 29, 1949–1955. 10.1200/JCO.2010.30.5037 21483002

[B48] Majumder S. Bhattacharjee A. Paul Chowdhury B. Bhattacharyya Majumdar S. Majumdar S. (2014). Antigen-pulsed CpG-ODN-activated dendritic cells induce host-protective immune response by regulating the T regulatory cell functioning in leishmania donovani-infected mice: critical role of CXCL10. Front. Immunol. 5:261. 10.3389/fimmu.2014.00261 24926293 PMC4044885

[B49] Matsumoto H. Thika A. A. Li H. Yeong J. Koo S.-L. Dent R. A.. (2016). Increased CD4 and CD8-positive T cell infiltrate signifies good prognosis in a subset of triple-negative breast cancer. Breast Cancer Res. Treat., 156, 237–247. 10.1007/s10549-016-3743-x 26960711

[B50] Ménard S. Casalini P. Campiglio M. Pupa S. Agresti R. Tagliabue E. (2001). HER2 overexpression in various tumor types, focussing on its relationship to the development of invasive breast cancer. Ann. Oncol. 12, S15–S19. 10.1093/annonc/12.suppl_1.S15 11521715

[B51] Meric F. Hung M. C. Hortobagyi G. N. Hunt K. K. (2002). HER2/neu in the managment of invasive breast cancer. J. Am. Coll. Surg. 194, 488–501. 10.1016/S1072-7515(02)01121-3 11949754

[B52] Mimura K. Ando T. Poschke I. Mougiakakos D. Johansson C. C. Ichikawa J. (2011). T cell recognition of HLA-A2 restricted tumor antigens is impaired by the oncogene HER2. Int. J. Cancer 128, 390–401. 10.1002/ijc.25613 20715101

[B53] Mittendorf E. A. Clifton G. T. Holmes J. P. Clive K. S. Patil R. Benavides L. C.. (2012). Clinical trial results of the HER-2/neu (E75) vaccine to prevent breast cancer recurrence in high-risk patients: from US Military Cancer Institute Clinical Trials Group Study I-01 and I-02. Cancer 118, 2594–2602. 10.1002/cncr.26574 21989902 PMC3428069

[B54] Mittendorf E. A. Holmes J. P. Ponniah S. Peoples G. E. (2008). The E75 HER2/neu peptide vaccine. Cancer Immunol. Immunother. 57, 1511–1521. 10.1007/s00262-008-0540-3 18536917 PMC11029853

[B55] Mittendorf E. A. Storrer C. E. Shriver C. D. Ponniah S. Peoples G. E. (2006). Investigating the combination of trastuzumab and HER2/neu peptide vaccines for the treatment of breast cancer. Ann. Surg. Oncol. 13, 1085–1098. 10.1245/ASO.2006.03.069 16865596

[B56] Morita M. Yamaguchi R. Tanaka M. Tse G. M. Yamaguchi M. Kanomata N.. (2016). CD8^+^ tumor-infiltrating lymphocytes contribute to spontaneous “healing” in HER2-positive ductal carcinoma *in situ*. Cancer Med. 5, 1607–1618. 10.1002/cam4.715 27061242 PMC4944888

[B57] Mortenson E. D. Fu Y. X. (2014). Anti-HER2/Neu passive-aggressive immunotherapy. Oncoimmunology 3:e27296. 10.4161/onci.27296 24605268 PMC3935925

[B58] Musolino A. Naldi N. Bortesi B. Pezzuolo D. Capelletti M. Missale G.. (2008). Immunoglobulin G fragment C receptor polymorphisms and clinical efficacy of trastuzumab-based therapy in patients with HER-2/neu-positive metastatic breast cancer. J. Clin. Oncol. 26, 1789–1796. 10.1200/JCO.2007.14.8957 18347005

[B59] Namjoshi P. Showalter L. Czerniecki B. J. Koski G. K. (2016). T-helper 1-type cytokines induce apoptosis and loss of HER-family oncodriver expression in murine and human breast cancer cells. Oncotarget. [Epub ahead of print]. 10.18632/oncotarget.10298 31666931 PMC6800266

[B60] O'Sullivan C. C. Smith K. L. (2014). Therapeutic considerations in treating HER2-positive metastatic breast cancer. Curr. Breast Cancer Rep. 6, 169–182. 10.1007/s12609-014-0155-y 25285186 PMC4180403

[B61] Pardoll D. M. (2012). The blockade of immune checkpoints in cancer immunotherapy. Nat. Rev. Cancer 12, 252–264. 10.1038/nrc3239 22437870 PMC4856023

[B62] Perez E. A. Ballman K. V. Tenner K. S. Thompson E. A. Badve S. S. Bailey H.. (2016). Association of stromal tumor-infiltrating lymphocytes with recurrence-free survival in the N9831 adjuvant trial in patients with early-stage HER2-positive breast Cancer. JAMA Oncol. 2, 56–64. 10.1001/jamaoncol.2015.3239 26469139 PMC4713247

[B63] Perez E. A. Thompson E. A. Ballman K. V. Anderson S. K. Asmann Y. W. Kalari K. R.. (2015). Genomic analysis reveals that immune function genes are strongly linked to clinical outcome in the North Central Cancer Treatment Group n(9831) Adjuvant Trastuzumab Trial. J. Clin. Oncol. 33, 701–708. 10.1200/JCO.2014.57.6298 25605861 PMC4334774

[B64] Pohlmann P. R. Mayer I. A. Mernaugh R. (2009). Resistance to trastuzumab in breast cancer. Clin. Cancer Res. 15, 7479–7491. 10.1158/1078-0432.CCR-09-0636 20008848 PMC3471537

[B65] Rakhra K. Bachireddy P. Zabuawala T. Zeiser R. Xu L. Kopelman A.. (2010). CD4^+^ T cells contribute to the remodeling of the microenvironment required for sustained tumor regression upon oncogene inactivation. Cancer Cell 18, 485–498. 10.1016/j.ccr.2010.10.002 21035406 PMC2991103

[B66] Rosalia R. A. Quakkelaar E. D. Redeker A. Khan S. Camps M. Drijfhout J. W.. (2013). Dendritic cells process synthetic long peptides better than whole protein, improving antigen presentation and T-cell activation. Eur. J. Immunol. 43, 2554–2565. 10.1002/eji.201343324 23836147

[B67] Rosenblatt J. Glotzbecker B. Mills H. Vasir B. Tzachanis D. Levine J. D.. (2012). PD-1 blockade by CT-011, anti PD-1 antibody, enhances *ex-vivo* T cell responses to autologous dendritic/myeloma fusion vaccine. J. Immunother. 34, 409–418. 10.1097/CJI.0b013e31821ca6ce 21577144 PMC3142955

[B68] Roses R. E. Paulson E. C. Sharma A. Schueller J. E. Nisenbaum H. Weinstein S.. (2009). HER-2/neu overexpression as a predictor for the transition from in situ to invasive breast cancer. Cancer Epidemiol. Biomarkers Prev. 18, 1386–1389. 10.1158/1055-9965.EPI-08-1101 19383888 PMC2713817

[B69] Saff R. R. Spanjaard E. S. Hohlbaum A. M. Marshak-Rothstein A. (2004). Activation-induced cell death limits effector function of CD4 Tumor-Specific T cells. J. Immunol. 172, 6598–6606. 10.4049/jimmunol.172.11.6598 15153474

[B70] Salgado R. Denkert C. Campbell C. Savas P. Nuciforo P. Aura C.. (2015a). Tumor-infiltrating lymphocytes and associations with pathological complete response and event-free survival in HER2-positive early-stage Breast Cancer treated with lapatinib and trastuzumab: a secondary analysis of the NeoALTTO trial. JAMA Oncol. 1, 448–454. 10.1001/jamaoncol.2015.0830 26181252 PMC5551492

[B71] Salgado R. Denkert C. Demaria S. Sirtaine N. Klauschen F. Pruneri G.. (2015b). The evaluation of tumor-infiltrating lymphocytes (TILs) in breast cancer: recommendations by an International TILs Working Group. Ann. Oncol. 26, 259–271. 10.1093/annonc/mdu450 25214542 PMC6267863

[B72] Scheuer W. Friess T. Burtscher H. Bossenmaier B. Endl J. Hasmann M. (2009). Strongly enhanced antitumor activity of trastuzumab and pertuzumab combination treatment on HER2-positive human xenograft tumor models. Cancer Res. 69, 9330–9336. 10.1158/0008-5472.CAN-08-4597 19934333

[B73] Sharma A. Koldovsky U. Xu S. Mick R. Roses R. Fitzpatrick E. (2012). HER-2 pulsed dendritic cell vaccine can eliminate HER-2 expression and impact ductal carcinoma in situ. Cancer 118, 4354–4362. 10.1002/cncr.26734 22252842 PMC3330145

[B74] Subbiah I. M. Gonzalez-Angulo A. M. (2014). Advances and future directions in the targeting of HER2-positive breast cancer: Implications for the future. Curr. Treat. Options Oncol. 15, 41–54. 10.1007/s11864-013-0262-4 24323591 PMC3933950

[B75] Tabuchi Y. Shimoda M. Kagara N. Naoi Y. Tanei T. Shimomura A.. (2016). Protective effect of naturally occurring anti-HER2 autoantibodies on breast cancer. Breast Cancer Res. Treat. 157, 55–63. 10.1007/s10549-016-3801-4 27113738

[B76] Teicher B. A. Doroshow J. H. (2012). The promise of antibody-drug conjugates. N. Engl. J. Med. 8, 1847–1848. 10.1056/NEJMe1211736 23134386

[B77] Tkach M. Coria L. Rosemblit C. Rivas M. A. Proietti C. J. Díaz Flaqué M. C.. (2012). Targeting Stat3 induces senescence in tumor cells and elicits prophylactic and therapeutic immune responses against breast cancer growth mediated by NK cells and CD4+ T cells. J. Immunol. 189, 1162–1172. 10.4049/jimmunol.1102538 22753933

[B78] Tran T. Diniz M. O. Dransart E. Gey A. Merillon N. Lone Y. C.. (2016). A therapeutic Her2/neu vaccine targeting dendritic cells preferentially inhibits the growth of low Her2/neu-expressing tumor in HLA-A2 transgenic mice. Clin. Cancer Res. 22, 4133–4144. 10.1158/1078-0432.CCR-16-0044 27006496

[B79] Triulzi T. De Cecco L. Sandri M. Prat A. Giussani M. Paolini B.. (2015). Whole-transcriptome analysis links trastuzumab sensitivity of breast tumors to both HER2 dependence and immune cell infiltration. Oncotarget 6, 28173–28182. 10.18632/oncotarget.4405 26334217 PMC4695052

[B80] Untch M. Fasching P. A. Konecny G. E. Hasmüller S. Lebeau A. Kreienberg R.. (2011). Pathologic complete response after neoadjuvant chemotherapy plus trastuzumab predicts favorable survival in human epidermal growth factor receptor 2-overexpressing breast cancer: results from the TECHNO trial of the AGO and GBG study groups. J. Clin. Oncol. 29, 3351–3357. 10.1200/JCO.2010.31.4930 21788566

[B81] Verma C. Eremin J. M. Robins A. Bennett A. J. Cowley G. P. El-Sheemy M. A.. (2013). Abnormal T regulatory cells (Tregs: FOXP3+, CTLA-4+), myeloid-derived suppressor cells (MDSCs: monocytic, granulocytic) and polarised T helper cell profiles (Th1, Th2, Th17) in women with large and locally advanced breast cancers undergoing neoadjuvant chemotherapy (NAC) and surgery: failure of abolition of abnormal treg profile with treatment and correlation of treg levels with pathological response to NAC. J. Transl. Med. 11:16. 10.1186/1479-5876-11-16 23320561 PMC3618083

[B82] Viehl C. T. Moore T. T. Liyanage U. K. Frey D. M. Ehlers J. P. Eberlein T. J.. (2006). Depletion of CD4+CD25+ regulatory T cells promotes a tumor-specific immune response in pancreas cancer-bearing mice. Ann. Surg. Oncol. 13, 1252–1258. 10.1245/s10434-006-9015-y 16952047

[B83] Vogel C. L. Cobleigh M. A. Tripathy D. Gutheil J. C. Harris L. N. Fehrenbacher L.. (2002). Efficacy and safety of trastuzumab as a single agent in first-line treatment of HER2-overexpressing metastatic breast cancer. J. Clin. Oncol. 20, 719–726. 10.1200/JCO.20.3.719 11821453

[B84] Wang K. Xu J. Zhang T. Xue D. (2016). Tumor-infiltrating lymphocytes in breast cancer predict the response to chemotherapy and survival outcome: a meta-analysis. Oncotarget 7, 44288–44298. 10.18632/oncotarget.9988 27329588 PMC5190096

[B85] Wesa A. K. Mandic M. Taylor J. L. Moschos S. Kirkwood J. M. Kwok W. W.. (2014). Circulating Type-1 Anti-Tumor CD4(+) T Cells are preferentially pro-apoptotic in Cancer patients. Front. Oncol. 4:266. 10.3389/fonc.2014.00266 25325015 PMC4178427

[B86] Witton C. J. Reeves J. Going J. J. Cooke T. G. Bartlett J. M. (2003). Expression of the HER1-4 family of receptor tyrosine kinases in breast cancer. J. Pathol. 200, 290–297. 10.1002/path.1370 12845624

[B87] Yakes F. M. Chinratanalab W. Ritter C. A. King W. Seelig S. Arteaga C. L. (2002). Herceptin-induced inhibition of phosphatidylinositol-3 kinase and Akt is required for antibody-mediated effects on p27, cyclin D1 and antitumor action. Cancer Res. 62, 4132–4141. 12124352

[B88] Zanardi E. Bregni G. de Braud F. Di Cosimo S. (2015). Better together: targeted combination therapies in Breast Cancer. Semin. Oncol. 42, 887–895. 10.1053/j.seminoncol.2015.09.029 26615133

[B89] Zhu X. Du L. Feng F. Ling Y. Xu S. (2014). Clinicopathological and prognostic significance of serum cytokine levels in breast cancer. *Clin*. Lab. 60, 1145–1151. 10.7754/clin.lab.2013.130738 25134383

